# Evaluating the Knowledge, Attitude, and Practice of Tuberculosis Among Health Sciences Students

**DOI:** 10.3390/healthcare13131534

**Published:** 2025-06-27

**Authors:** Alvin F. Terry, İlker Etikan

**Affiliations:** Department of Biostatistics, Faculty of Medicine, Near East University, Near East Avenue, North Cyprus, 99138 Mersin 10, Turkey; ilker.etikan@neu.edu.tr

**Keywords:** tuberculosis, knowledge, attitude, practice, public health, students, pharmacy, medical

## Abstract

**Background:** Liberia is among the 30 countries with a high burden of tuberculosis worldwide. Health sciences students, who are future health professionals, have essential roles in curtailing the spread of TB. This study aims to evaluate the knowledge, attitude, and practice (KAP) of tuberculosis (TB) among health sciences students. **Methods:** This study used a quantitative cross-sectional design to assess Medical, Pharmacy, and Public Health students at the University of Liberia’s KAP regarding TB using a modified previously used self-administered questionnaire reviewed by subject experts from 1 April 2025 to 23 April 2025. SPSS 26 was used for analysis. Descriptive statistics, Mann–Whitney, Kruskal–Wallis, and multivariate logistic regression tests were used for analysis. **Results:** In total, 630 students participated, of which 51.7% were females, 83% were aged 24 or above, 81.6% were single, and 96.7% had never smoked. The KAP levels were 65.9%, 97.3%, and 94.8%, respectively. Higher TB knowledge was significantly associated with being enrolled in the Medical program (OR = 2.20, 95% CI: 1.28–3.76, *p* < 0.05), being in year 4 and 5 (OR = 1.79, 95% CI: 1.09–2.98, *p* < 0.05; OR = 2.28, 95% CI: 1.08–4.78, *p* < 0.05), being unemployed (OR = 1.58, 95% CI: 1.09–2.31, *p* < 0.05), and having personal acquaintance with individuals diagnosed with TB (OR = 1.64, 95% CI: 1.11–2.42, *p* < 0.05). **Conclusions:** The knowledge level among students was good. They had a positive attitude, and their practice levels were good. However, gaps remain in understanding latent TB and proper disinfection methods for TB-related materials. Strengthening the health curriculum to address these specific knowledge gaps is recommended to better align students’ knowledge with their attitudes and practices.

## 1. Introduction

Tuberculosis (TB) is a contagious bacterial illness that primarily targets the lungs (pulmonary TB) and is transmitted from infected individuals by airborne bacterial droplets [1,2]. One out of four people living today has asymptomatic *Mycobacterium tuberculosis*, which is latent TB and is not transmissible [3]. Mycobacterium tuberculosis infection increases the lifetime risk of active TB by 10% [3]. Immunocompromised (HIV, underweight, diabetic, and tobacco users) people are at higher risk [1,3]. Since it was discovered in 1882, TB has caused the deaths of over 1 billion people [4,5]. This number is higher than malaria, smallpox, HIV/AIDS, cholera, plague, and influenza deaths together [5]. In 2023, around 8.2 million individuals received a new diagnosis of TB, with a higher prevalence among males (55%), followed by females (33%) and youth (12%) [6]. Additionally, in 2023, 1.25 million individuals—including 161,000 with HIV—lost their lives to TB [1]. In 2014 and 2015, attempting to end the TB pandemic by 2035, the WHO’s End TB Strategy was endorsed by the United Nations (UN) and the World Health Organization (WHO) [7]. The TB incidence rate from 2015 to 2023 was 8.3%, significantly below the WHO End TB Strategy target of reducing TB in 2025 by 50% [8]. This highlights the need for concerted efforts in combating this deadly disease. To understand the global trend, assessing country-level progress, particularly in high-burden, low-resource settings like Liberia, where the incidence and drug-resistant TB remains a concern, is essential. By 2023, the national TB incidence was 308 cases per 100,000 persons [9], positioning Liberia among the 30 nations with the highest tuberculosis burden worldwide [8]. In late 2021, 260 individuals were receiving treatment for drug-resistant TB at the tuberculosis annex in Liberia [10]. These statistics highlight the urgent need to strengthen TB control initiatives, particularly through training healthcare students, who are the future healthcare providers. The knowledge, attitude, and practice (KAP) research technique has provided valuable insights for curbing TB, as diminished KAP scores correlated with postponed Medical care seeking behavior [11]. A review of studies across multiple regions reveals varying levels of tuberculosis-related knowledge, attitudes, and practices (KAP) among students in both health and non-health disciplines. Students in health-related fields demonstrated higher KAP scores than their non-health counterparts, as reported in studies from Saudi Arabia and Pakistan [12,13]. In the Middle East, healthcare students in Jordan exhibited satisfactory knowledge, attitude, and practice (KAP) levels [14]. In contrast, a study in Afghanistan reported inadequate TB KAP among both healthcare and non-healthcare students [15]. In South Asia, research from India found moderate knowledge but poor attitudes and practices among nursing students [16]. In contrast, final-year Medical trainees in India, Canada, and Uganda showed adequate knowledge and practical competencies [17]. In Western contexts, such as the United States and the United Kingdom, studies found that health profession students generally have satisfactory knowledge, but highlight areas needing improvement in attitudes and confidence [18,19]. Collectively, these findings underscore the global variability in TB-related KAP and point to the need for more targeted, context-specific educational interventions.

Liberia needs to strengthen TB prevention and control measures, with timely diagnosis being critical to containing this communicable disease. While international funding, including support from agencies like USAID, has been significant [20], sustainable TB control ultimately depends on the country’s own commitment to education, healthcare infrastructure, and public health initiatives, such as this research targeting future healthcare workers. To prevent and manage TB, healthcare providers and professionals play crucial roles [21,22]. Students, especially in health sciences, must learn enough about TB and change their attitudes and behaviors to graduate with the information, skills, and attitudes needed to manage tuberculosis [23]. No studies have been conducted in Liberia that have assessed the KAP of health sciences students concerning TB. Evaluating university students’, especially health sciences students’, awareness about tuberculosis and identifying knowledge gaps is essential for informing future health educational initiatives. This assessment will assist school administrators, health administrators, health authorities, and policymakers in designing effective and targeted preventive programs to address communicable diseases, including TB [24]. Therefore, this study aims to fill a critical knowledge gap by assessing the TB-related knowledge, attitudes, and practices (KAP) of Medical, Pharmacy, and Public Health students at the University of Liberia.

## 2. Methods

### 2.1. Study Design and Setting

This study employed a quantitative cross-sectional design at the University of Liberia’s College of Health Sciences (Medical, Pharmacy, and Public Health). The University of Liberia, established in 1862 as Liberia College, attained university status in 1951. Its College of Health Sciences comprises four schools—Medicine, Pharmacy, Public Health, and Midwifery—located in Fendell and Monrovia, Montserrado County. The School of Medicine, founded in 1968, admits approximately 50 students annually; the School of Pharmacy, established in 1986, has a similar intake. The School of Public Health, established in 2018, enrolls approximately 200 students annually. The duration of study is five years for Medicine, and four years for both Pharmacy and Public Health programs. Tuberculosis education is integrated theoretically within these curricula through courses such as Infectious and Chronic Diseases [25,26,27,28]. The study setting was unique because the Medical and Pharmacy schools are the only ones in Liberia. At the same time, the Public Health program has the highest enrollment compared to other universities’ Public Health programs.

### 2.2. Data Collection Procedure

The study received ethical approval from the Near East University Scientific Research Ethical Committee (NEU/2025/131-1934). Participants were recruited through stratified random sampling by program (Medicine, Pharmacy, and Public Health) and year of study. A structured, self-administered hard-copy questionnaire was completed by participating students after their regular classes and after hospital meetings for Medical and Pharmacy students on rotation at the hospitals. It took approximately 20 min to complete the questionnaire. Informed consent was obtained before participation. Data collection lasted from 1 April 2025 to 23 April 2025. To ensure anonymity and confidentiality, the questionnaire contained no personal identifiers.

### 2.3. Criteria for Selecting the Study Sample

The inclusion criteria were students who were Liberian nationals, as almost all students are Liberian, aged 18 years and above, and enrolled in the Medical, Pharmacy, and Public Health program for the first Semester of 2024/2025. Students from other departments were excluded.

### 2.4. Sampling Size Calculation and Sampling Technique

The sample size was calculated using G*Power 3.1.9.7 [29], assuming a medium effect size (f = 0.25), 95% power, and a 0.05 significance level for the F-test, yielding a minimum of 300 participants. To account for non-response and ensure adequate power for subgroup analyses, 686 students were recruited. Forty-two students were excluded for providing incomplete responses, and fourteen for not being aware of TB. Six hundred and thirty responses were included in the final analysis. Stratified random sampling was employed, using academic discipline (Medical, Pharmacy, and Public Health) and level of study as strata.

### 2.5. Data Collection Instrument

The data collection tool used was a structured, closed-ended questionnaire adapted from previous KAP studies of tuberculosis [30,31] with a similar population, minimal modifications, and reviewed by subject experts to ensure content validity, relevance, and clarity for the target population. The reliability of the instrument was assessed using Cronbach’s alpha. The subscale alphas ranged from 0.73 to 0.79, with an overall reliability coefficient of 0.82, indicating good internal consistency. The questionnaire comprised four sections. Section 1 captured demographic information, including gender, age, marital status, study program and level, employment status, funding source, smoking and alcohol use, residence type, family socioeconomic status, and TB-related exposure (e.g., knowing someone with TB and sources of TB information). Section 2 assessed knowledge with 10 items using True/False/I do not know response options. Section 3 measured attitudes with 10 Likert-scale items (strongly agree to strongly disagree). Section 4 evaluated practices with seven items rated on a five-point frequency scale (always to never).

### 2.6. Scoring of the Study Questionnaire

For the knowledge questions, 1 point was given for ‘True’, 0 points for ‘False’, and 0 points for ‘I do not know’. Question 8 was recoded. The sum was taken to obtain the total knowledge score, with scores ranging from 0 to 10. The attitude questions were scored as follows: 1: strongly disagree, 2: disagree, 3: neutral, 4: agree, and 5: strongly agree. Questions 2, 5, and 9 were recoded. The sum was taken, and the score ranged from 10 to 50. Practice questions were rated on a scale of 1 to 5, with “always” receiving 5, “usually” receiving 4, “sometimes” receiving 3, “rarely” receiving 2, and “never” receiving 1. The total scores range from 7 to 35. The KAP of TB was organized into two categories based on the Bloom cut-off score [32,33], where 60% or more of the respective total score was considered “adequate knowledge” (≥6), “positive attitude” (≥30), and “good practice” (≥21).

### 2.7. Statistical Analysis

The data collected was each numbered. It was then manually entered into Excel and checked with the number as a data quality check. After the quality check, it was loaded into SPSS 26 for analysis. A normality check was performed using the Shapiro–Wilk test and the Kolmogorov–Smirnov test. Descriptive statistics such as frequency and percentages were generated for the participants’ sociodemographic variables and the categorization of the knowledge, attitude, and practice level. Because the study data were not normally distributed, non-parametric tests, such as the Kruskal–Wallis and Mann–Whitney U tests, were used to find the association between sociodemographic variables and the total continuous scores of knowledge, attitude, and practice. A *p*-value of <0.05 was considered statistically significant. Spearman’s rank correlation was used to find the association between the continuous KAP TB total score. A *p*-value of <0.05 was considered statistically significant. After categorizing the KAP score, univariate logistic regression with a *p*-value of <0.05 was included in the multivariate logistic regression. The multivariate logistic regression was used to find factors associated with KAP.

## 3. Results

### 3.1. Demographic Characteristics of the Study Respondents and Knowledge, Attitude, and Practice (KAP) Scores (n = 630)

Table 1 shows the demographic characteristics of the study participants. Over half of the participants were female (*n* = 326; 51.7%), with the majority (*n* = 523; 83%) being aged 24 years or older. Most were single (*n* = 514; 81.6%). More students were from the Public Health program (*n* = 373; 59.2%), and year 4 students comprised the highest percentage (*n* = 171; 27.1%) in the study. Most were unemployed (365; 57.3%). Regarding sponsorship (*n* = 222; 35.2%) were sponsored by parents/guardians. Almost all participants had never smoked (*n* = 609; 96.7%), and more than half drank occasionally (*n* = 334; 53%). Most live at home with their family (*n* = 391; 62.1%) and are from low-income families (*n* = 330; 52.4%). Most did not know people with TB (*n* = 360; 57.1%), and the sources from which participants heard about TB are listed in Table 1. In Table 1, the non-parametric Kruskal–Wallis and Mann–Whitney U tests are used for comparison between the continuous KAP and demographic variables, as explained in Section 2.6. There is a statistically significant difference between gender, department, type of residence (*p* < 0.05), and participants’ knowledge and attitude regarding TB score. Age, marital status, employment, knowing those with TB, and source of hearing about TB show statistically significant differences (*p* < 0.05) to the knowledge score. Statistically significant differences (*p* < 0.05) exist between students’ year of study, mode of sponsorship, and their knowledge and practice scores.

### 3.2. Respondent TB Knowledge, Attitude, and Practice Level

Table 2 shows the descriptive statistics of the study participants who have heard about TB KAP levels. The adequate TB knowledge score among those who have heard about TB is 65.9%, while for positive attitude it is 97.3%, and for good practice it is 94.8%, as described in Section 2.5.

### 3.3. Assessing Respondent TB Knowledge

Table 3 displays the frequency of the participants who have heard about TB response to TB knowledge questions. Tuberculosis (TB), a communicable disease, is caused by Mycobacterium tuberculosis (K1); this had the highest correct response of 87.6%. TB spreads mainly through the respiratory tract, like through coughing and sneezing; this had the second highest correct response (K2) (82.9%). In contrast, those with latent tuberculosis are not considered active TB cases and do not transmit the disease; this had the lowest correct response (K3) (24.9%). Items used by TB patients can be disinfected by drying them in strong sunlight for at least half an hour (K8); this had the second lowest correct response (28.3%).

### 3.4. Assessing Respondent TB Attitude

Table 4 shows the frequency of participants who have heard about TB response to the TB attitude questions. The question with the highest percentage for “strongly agree” was “I would encourage anyone around me with TB to seek Medical treatment promptly” (A3) (80.5%). Additionally, 77.8% strongly agree that, should they develop tuberculosis, it is important to quickly alert their doctor and/or loved ones (A1). The lowest percentage for “strongly agree” was A9, “If I had TB, not being able to leave work or not knowing where to seek care would be reasons for not visiting a healthcare facility” (24.8%), with the majority remaining neutral in their response (35.4%).

### 3.5. Assessing Respondent TB Practice

Table 5 shows the frequency of the participants who have heard about TB response to TB practice questions. In total, 71.4% of the respondents said that, for fear of spreading infections, they always cover their mouths when they cough or sneeze (P1). More than half (57.6%) said that after leaving the hospital, they clean their hands with soap or hand sanitizer (P3). They also agree that they would seek Medical attention at a health center if their cough continued for more than two weeks, for examination (P6) (51.6%). The least practiced item was reading educational materials that raise awareness about tuberculosis (35.7%).

### 3.6. Correlation Between Knowledge, Attitude, and Practice (n = 630)

Table 6 shows Spearman’s correlation between KAP of the study respondents who knew about TB. Knowledge had a moderate positive statistical correlation with attitude (r = 0.383, *p* < 0.001) and a weak to moderate positive significant correlation with practice (r = 0.330, *p* < 0.001). Additionally, attitude showed a weak, statistically significant positive correlation with practice (r = 0.224, *p* < 0.001).

### 3.7. Assessing Respondents’ Knowledge Level of TB and Sociodemographic Variables

Significant variables (*p* < 0.05) in the univariate logistic regression were included in the multivariate logistic regression. As shown in Table 7, the analysis revealed that several demographic factors were associated with higher tuberculosis (TB) knowledge. Being a Medical student (OR = 2.20, 95% CI: 1.28–3.76, *p* < 0.05), in the 4th year (OR = 1.79, 95% CI: 1.08–2.98, *p* < 0.05) or 5th year of study (OR = 2.28, 95% CI: 1.08–4.78, *p* < 0.05), unemployed (OR = 1.58, 95% CI: 1.09–2.31, *p* < 0.05), or personally knowing someone with TB (OR = 1.64, 95% CI: 1.11–2.42, *p* < 0.05) were significantly associated with greater odds of having good TB knowledge. In contrast, being female (OR = 0.62, 95% CI: 0.43–0.91, *p* < 0.05) or sponsored by a parent or guardian (OR = 0.56, 95% CI: 0.37–0.84, *p* < 0.05) were associated with lower odds of good TB knowledge. Attitude and practice were not associated with demographic variables in the univariate logistic regression.

## 4. Discussion

This is the first study in Liberia to examine tuberculosis-related knowledge, attitudes, and practices (KAP) among health sciences students at the University of Liberia. While 65.9% of students had good knowledge, attitudes (97.3%), and practices (94.8%) were notably higher. However, gaps were observed in understanding latent tuberculosis (TB) and the disinfection of TB-related materials. Moderate knowledge, positive attitude, and practice demonstrated that behaviors can be driven by witnessing real-life instances, clinical exposure, and societal norms rather than theoretical knowledge. In addition, many students in Liberia witnessed or heard about the devastating effects of Ebola [34], which may explain why attitudes and practices are high, as having a positive attitude and good practices serve as preventive measures for other diseases besides TB. Additionally, participants might have tended to overstate their attitudes and practices, as the study was a self-administered questionnaire. In Liberia, which is among the 30 nations with the most significant burden of tuberculosis [8], the knowledge level gap is alarming, as these students are future healthcare providers and will play a cardinal role in detecting, treating, and providing Public Health education regarding TB. This study result is similar to final-year Medical students in Iran, Canada, India, Uganda, and Italy, in which moderate knowledge and positive attitudes were found [17,35,36,37]. This study, in contrast, found that practice was poor. Most final-year Medical students in Karachi display good KAP of TB [34]. Although using different study instruments, the similar study populations might account for similar results. Most participants in a KAP study at Taif University demonstrated good TB knowledge [30]; their attitudes and practices were relatively low. Both Medical students and students from other departments in Indonesia demonstrated moderate knowledge [38]. These studies were conducted among Medical students and students from non-health backgrounds, unlike this study, which was exclusively among health students, which might be the reason for the differences in results. Additionally, the study methodology and disparities in ethnic, regional, and cultural features across the people tested possibly explain the differences observed. Pharmacy students at the University of Wolverhampton display acceptable TB knowledge [19]. The authors of [30] found a low level of TB knowledge among Medical and health undergraduate students. Even though the study setting differs, the results are significant as both sets of participants were health sciences students. Higher age, being from the Medical department, and being in the fourth and fifth years were associated with a higher and significant knowledge level in this study (*p* < 0.05). In Saudi Arabia and Indonesia [39,40], researchers found a knowledge difference based on gender, with women having a higher level of knowledge, which is in contrast to this study, where males have a higher level of knowledge. Long-standing cultural practices, which sometimes require women to be pressured to marry and have children, thereby delaying their studies, are an ongoing endeavor in Liberia, explaining the differences in this study. No gender and college differences were found in [41]. Additionally, students of higher ages [42], higher academic years, and from the Medical department had more TB-related knowledge [30,39,42,43,44]. Like many other nations, Liberia follows a curriculum where basic Medical and Public Health ideas are introduced at a lower level and taught in detail as the students advance in class. Students in higher years, typically starting from year 3 in Liberia, have been exposed to more advanced subjects and commence clinical rotations, unlike year 1 and 2 students. As the number of years at university increases, so does the student age, which accounts for the differences.

This study observes that being unemployed, self-funded, and knowing people who had or have TB was associated with higher TB knowledge (*p* < 0.05). Unemployed students have extra time and may have fewer external obligations, which gives them more time to engage with their subject matter and engage in other activities like disease awareness in communities, especially for diseases like TB, a public health concern in Liberia. Scholarship students are expected to maintain and meet grade requirements, and self-funded students know the burden and financial loss associated with failure, so they devote more time to study, thereby having improved knowledge. When you are connected with someone who has a disease condition, it results in increased awareness and engagement. You engage more with the topic, thereby resulting in increased knowledge [40,45].

In the univariate logistic regression, no statistically significant difference was observed in the study participants’ attitudes and practices regarding tuberculosis and demographic characteristics, with almost all participants having positive attitudes and good practice scores. Following the devastating effects of the Ebola crisis in Liberia, emphasis has been placed on infectious disease prevention [46], apparently resulting in the positive attitude and good practice observed in this study. Additionally, following the COVID-19 global pandemic, there has been a heightened global awareness of preventive measures. It is also possible that, considering their field of study and expectations from them, they might have inflated their attitudes and practices, which is possible due to the survey nature of the study. The authors of [34] found no differences in gender and TB attitude and practice among Medical students in Iran. In Malaysia, no differences were found in KAP of TB by year of study, age, or gender, but a difference was observed by school or department [47]. Additionally, [48] found differences in attitudes toward TB, sex, and year of education among immigrants in Sweden.

This study found a statistically significant correlation between knowledge, attitudes, and practices regarding tuberculosis (TB) among health sciences students at the University of Liberia. This suggests that students with more knowledge were more inclined to demonstrate favorable attitudes and participate in suitable preventative measures. The findings support the Health Belief Model [49], which suggests that students with more knowledge and an understanding of their susceptibility to the disease will develop a more positive attitude and adopt good practices. People’s perceptions about their vulnerability, perceived advantages, and self-efficacy significantly correlate with their adoption and use of preventive measures for prevalent curable illnesses [50]. Similarly, other studies reported a statistically significant correlation between knowledge, attitude, and practice [14,31,43,51]. The authors of [47] reported a statistically significant correlation of knowledge with attitude and practice, but not between attitude and practice. This study must be viewed in the context of global financial issues. USAID-supported TB projects in several countries have been discontinued, disrupting diagnostic services, drug supply chains, and health worker assistance [20,52,53]. These external difficulties underscore the need to enhance local capacity through training, education, and behavior change, particularly among health sciences students who will play crucial roles in TB control. Additionally, the gap identified in this study highlights the need for an improved curriculum that integrates both theoretical concepts and clinical exposure. By strengthening TB education in health sciences, this study supports SDG 3, specifically target 3.3, which aims to end the TB epidemic by 2030. Equipping future healthcare workers with accurate and comprehensive TB knowledge is essential for early diagnosis, effective treatment, and community-level prevention.

By assessing the knowledge, attitudes, and practices (KAP) of tuberculosis among health sciences students (Medical, Pharmacy, and Public Health), this study has technically identified gaps in national TB control efforts and provided baseline data for use by school administrators, government, health ministry, and the Public Health Institute. Its large sample size of 630 provides statistical power, which is generalizable to the study population, and is a strength of this study. However, it is essential to note that the limitation of this study lies in its self-reported questionnaire nature, which may have resulted in students overreporting their KAP and making it non-generalizable to other settings. This study is also limited by the low number of Pharmacy students who consented to participate. The voluntary nature of the study might have also led to biased samples. Future research should include students from other universities’ health sciences colleges, and equal samples should be included from the study population. A mixed-method study design is also recommended.

## 5. Conclusions

This study found that the knowledge level was good among the majority, and the attitudes and practices of the health sciences students (Medical, Pharmacy, and Public Health) at the University of Liberia were positive and good. However, a considerable knowledge gap was seen in their understanding of latent TB and disinfection of TB-related materials. These results highlight the need to enhance health-related curriculum to increase students’ knowledge and guarantee that knowledge fits the reported attitudes and practices observed in the study. More interactive teaching techniques, workshops, practical experience, and hands-on demonstrations should be considered for newly admitted students.

## Figures and Tables

**Table 1:** Demographic characteristics of the study respondents and KAP scores (*n* = 630).

Variables	*n* (%)	Knowledge Scores		Attitude Scores		Practice Scores	
		Mean Rank	*p* *	Mean Rank	*p* *	Mean Rank	*p* *
Total	630						
**Sex**			**<0.001**		**<0.005**		0.912
Male	304 (48.3)	357.11		338.04		314.67	
Female	326 (51.7)	276.70		294.48		316.27	
**Age**			**<0.001**		0.116		0.078
18–20	33 (5.2)	234.41		342.53		286.02	
21–23	74 (11.7)	253.30		276.89		276.69	
24 and above	523 (83.0)	329.42		319.26		322.85	
**Marital Status**			**<0.05**		0.678		0.402
Single	514 (81.6)	305.52		316.92		312.62	
Married	116 (18.4)	359.72		309.19		328.25	
**Department**			**<0.001**		**<0.001**		0.891
Public Health **	373 (59.2)	277.58		296.71		314.95	
Medical	202( 32.1)	365.00		353.43		319.08	
Pharmacy	55 (8.7)	390.90		303.61		306.08	
**Year of Study**			**<0.05**		0.800		**<0.05**
1.0	138 (21.9)	283.42		302.33		317.41	
2.0	129 (20.5)	298.41		315.13		276.93	
3.0	109 (17.3)	331.96		330.10		307.10	
4.0	171 (27.1)	320.86		313.00		334.19	
5.0	83 (13.2)	362.72		323.95		344.79	
**Employment**			**<0.05**		0.17		0.77
Employed/Self-Employed	265 (42.1)	291.77		303.86		317.98	
Unemployed	365 (57.9)	332.73		323.95		313.70	
**Mode of Sponsorship**			**<0.001**		0.062		**<0.05**
Self-Funded	298 (47.3)	322.31		316.61		324.92	
Scholarship	110 (17.5)	384.02		347.77		340.04	
Parent/Guardian	222 (35.2)	272.41		298.02		290.70	
**Smoking History**			0.608		0.452		0.342
Former	21 (3.3)	295.60		286.21		278.52	
Never Smoke	609 (96.7)	316.19		316.51		316.78	
**Alcohol Consumption**			0.125		0.068		0.117
Never	296 (47.0)	303.74		301.47		327.55	
Occasional	334 (53.0)	325.92		327.93		304.82	
**Type of Residence**			**<0.05**		**<0.001**		0.08
Dormitory/Renting Privately	239 (37.9)	337.93		349.88		299.50	
Home with Family	391 (62.1)	301.79		294.48		325.28	
**Family Economic Status**			0.49		0.78		0.77
Low Income	330 (52.4)	320.22		317.40		317.49	
Middle Income	300 (47.6)	310.31		313.41		313.31	
**Where did you hear about TB?**			0.07		0.61		0.18
Print Media, Radio Stations, TV Program, or Social Networks	118 (18.7)	275.82		287.98		315.12	
Print Media, Radio Stations, TV Programs, Social Networks, Family Members, Colleagues, Neighbours, Health professionals, or Colleagues	196 (31.1)	311.06		324.99		324.62	
Print Media, Radio Stations, TV Programs, or Social Networks, Family Members, Colleagues, Neighbours, Health professionals, Colleagues, Booklets, Flyers, Lessons, or Other Things that are Printed	104 (16.5)	349.98		325.64		311.51	
Print Media, Radio Stations, TV Program, Social Networks, Booklets, Flyers, Lessons, or Other Things that are Printed	29 (4.6)	359.71		319.02		388.28	
Family Members, Colleagues, Neighbours, Health professionals, or Colleagues	82 (13.0)	314.27		314.94		309.77	
Family Members, Colleagues, Neighbours, Health professionals, Colleagues, Booklets, Flyers, Lessons, or Other Things that are Printed	78 (12.4)	328.69		327.31		295.48	
Booklets, Flyers, Lessons, or Other Things that are Printed	23 (3.7)	304.98		287.46		254.30	
**Knowing TB People**			**<0.001**		0.420		0.654
No	360 (57.1)	282.82		310.45		312.70	
Yes	270 (42.9)	359.08		322.23		319.24	

** Public Health includes students majoring in Environmental Health, Health Systems Management, and Applied Epidemiology. * Mann–Whitney U test or Kruskal–Wallis. *p*-value in bold indicates statistical significance

**Table 2:** Descriptive statistics of study respondents’ KAP of TB levels (*n* = 630).

KAP Component	Levels	*n*	Median (IQR *)	Percent
Knowledge	Inadequate	215	4.0 (3–5.0)	34.1
	Adequate	415	7.0 (6.9–8.0)	65.9
Attitude	Negative	17	24.0 (22–25.0)	2.7
	Positive	613	44.0 (41–46.0)	97.3
Practice	Poor	33	19.0 (16.5–20.0)	5.2
	Good	597	29.0 (26–33.0)	94.8

* IQR = Interquartile Range.

**Table 3:** The questions related to the frequency of study respondents’ knowledge of TB among those who heard about TB (*n* = 630).

Knowledge Questions	True *n* (%)	False/Do Not Know *n* (%)
K1. Tuberculosis (TB), a communicable disease, is caused by Mycobacterium tuberculosis.	552 (87.6)	78 (12.4)
K2. TB spreads mainly through the respiratory tract, like through coughing and sneezing.	522 (82.9)	108 (17.1)
K3. Those with latent tuberculosis are not considered active TB cases and do not transmit the disease.	157 (24.9)	473 (75.1)
K4. Coughing for more than two weeks or hemoptysis is a common sign that someone might have TB. Quick Medical consultation and treatment are needed.	504 (80.0)	126 (20.0)
K5. The standard Medical treatment plan for TB patients should be at least 6 months, and should be changed accordingly by considering the condition of TB and drug resistance.	482 (76.5)	148 (23.5)
K6. Close Contacts of TB cases who test negative initially should be retested after six months and one year.	377 (59.8)	253 (40.2)
K7. Bacillus Calmette-Guérin (BCG) vaccination can protect children from TB infection.	375 (59.5)	255 (40.5)
K8. Items used by TB patients can be disinfected by drying them in strong sunlight for at least half an hour.	178 (28.3)	452 (71.7)
K9. Disinfection by boiling and high-pressure steam is the best method for killing TB bacteria. Boiling should continue for more than 10 min.	276 (43.8)	354 (56.2)
K10. TB risk factors: HIV infection, history of tuberculosis exposure, immune weakness, etc.	501 (79.5)	129 (20.5)

**Table 4:** The questions related to the frequency of study participants’ attitudes toward TB among those who heard about TB (*n* = 630).

Attitude Toward Tuberculosis Questionnaire	Strongly Agree, *n* (%)	Agree *n* (%)	Neutral *n* (%)	Disagree *n* (%)	Strongly Disagree *n* (%)
A1. Should I develop tuberculosis, it is important to quickly alert my doctor and/or loved ones.	490 (77.8)	97 (15.4)	25 (4.0)	12 (1.9)	6 (1.0)
A2. Access to TB screening services is limited at many health facilities, including community health centers.	190 (30.2)	204 (32.4)	188 (29.8)	35 (5.6)	13 (2.1)
A3. I would encourage anyone around me with TB to seek Medical treatment promptly.	507 (80.5)	92 (14.6)	17 (2.7)	8 (1.3)	6 (1.0)
A4. TB is considered a serious illness.	462 (73.3)	93 (14.8)	59 (9.4)	12 (1.9)	4 (0.6)
A5. Yearly health check-ups cannot prevent TB.	212 (33.7)	92 (14.6)	304 (48.3)	15 (2.4)	7 (1.1)
A6. There is an urgent need for education about tuberculosis.	403 (64.0)	112 (17.8)	83 (13.2)	15 (2.4)	17 (2.7)
A7. Isolating TB patients may help reduce the transmission of tuberculosis.	326 (51.7)	163 (25.9)	100 (15.9)	25 (4.0)	16 (2.5)
A8. Controlling TB requires active participation from the public.	341 (54.1)	164 (26.0)	84 (13.3)	10 (1.6)	31 (4.9)
A9. If I had TB, not being able to leave work or not knowing where to seek care would be reasons for not visiting a healthcare facility.	156 (24.8)	223 (35.4)	216 (34.3)	16 (2.5)	19 (3.0)
A10. If diagnosed with TB, I would follow my treatment plan exactly as directed by my physician.	440 (69.8)	106 (16.8)	40 (6.3)	27 (4.3)	17 (2.7)

**Table 5:** The questions related to the frequency of study participants’ practice regarding TB among those who heard about TB (*n* = 630).

Practice Regarding Tuberculosis	Always *n* (%)	Usually *n* (%)	Sometimes *n* (%)	Rarely *n* (%)	Never *n* (%)
P1. For fear of spreading infections, I always cover my mouth when I cough or sneeze.	450 (71.4)	119 (18.9)	54 (8.6)	5 (8.0)	2 (0.3)
P2. I eat a diet that is nutritious and well-balanced to remain healthy and prevent illnesses.	262 (41.6)	119 (18.9)	212 (33.7)	28 (4.4)	9 (1.4)
P3. After leaving the hospital, I clean my hands with soap or hand sanitizer.	363 (57.6)	133 (21.1)	115 (18.3)	14 (2.2)	5 (0.8)
P4. I exercise regularly to maintain my health.	226 (35.9)	119 (18.9)	214 (34.0)	59 (9.4)	12 (1.9)
P5. I wear a facemask when visiting a hospital.	248 (39.4)	108 (17.1)	203 (32.2)	44 (7.0)	27 (4.3)
P6 I would seek Medical attention at a health center if my cough continued for more than two weeks, for examination.	324 (51.4)	171 (27.1)	95 (15.1)	31 (4.9)	9 (1.4)
P7. I read educational materials that raise awareness about tuberculosis.	225 (35.7)	121 (19.2)	196 (31.1)	54 (8.6)	34 (5.4)

**Table 6:** Correlation between knowledge, attitude, and practice (*n* = 630).

Variable	Knowledge	Attitude	Practice
Knowledge	1	r = 0.383 (*p* < 0.001)	r = 0.330 (*p* < 0.001)
Attitude		1	r = 0.224 (*p* < 0.001)
Practice			1

**Table 7:** Comparison of the level of TB knowledge with sociodemographic variables.

Variables	Odds Ratio (95% Confidence Interval)	*p*
**Knowledge**		
**Age**		
18–20	Reference	
21–23	1.61 (0.67–3.86)	0.28
24 and above	1.23 (0.56–2.70)	0.60
**Sex**		
Male	Reference	
Female	0.62(0.43–0.91)	**<0.05**
**Marital Status**		
Single	Reference	
Married	1.60 (0.94–2.71)	0.08
**Study Program**		
Public Health **	Reference	
Medicine	2.20 (1.28–3.76)	**<0.05**
Pharmacy	1.85 (0.85–3.99)	0.12
**Year of Study**		
1	Reference	
2	1.12 (0.66–1.90)	0.67
3	1.43 (0.81–2.51)	0.22
4	1.79 (1.08–2.98)	**<0.05**
5	2.28 (1.08–4.78)	**<0.05**
**Employment**		
Employed/Self-Employed	Reference	
Unemployed	1.58 (1.09–2.31)	**<0.05**
**Mode of Sponsorship**		
Self-Funded	Reference	
Scholarship	1.08 (0.55–2.09)	0.82
Parent/Guardian	0.56 (0.37–0.84)	**<0.05**
**Residence**		
Dormitory/Renting Privately	Reference	
Home with Family	1.08 (0.72–1.64)	0.70
**Knowing TB People**		
No	Reference	
Yes	1.64 (1.11–2.42)	**<0.05**

** Public Health includes students majoring in Environmental Health, Health System Management, and Applied Epidemiology. *p*-value in bold indicates statistical significance.

## Data Availability

Data are available from the corresponding author upon reasonable request.
